# Cochrane corner: convalescent plasma or hyperimmune immunoglobulin for people with COVID-19

**DOI:** 10.11604/pamj.supp.2020.35.24157

**Published:** 2020-06-16

**Authors:** Chukwudi Arnest Nnaji, Charles Shey Wiysonge

**Affiliations:** 1 Cochrane South Africa, South African Medical Research Council, Cape Town, South Africa; 2 School of Public Health and Family Medicine, University of Cape Town, Cape Town, South Africa; 3 Centre for Evidence-based Health Care, Division of Epidemiology and Biostatistics, Stellenbosch University, Cape Town, South Africa

**Keywords:** Coronavirus, COVID-19, convalescent plasma, hyperimmune immunoglobulin, transfusion

## Abstract

As the novel coronavirus continues to spread globally and across Africa, efforts are being accelerated to identify effective preventive and therapeutic measures to mitigate its burden. Convalescent plasma and hyperimmune immunoglobulin are being considered as potential therapeutic options for the coronavirus disease 2019 (COVID-19). We highlight and contextualize the findings of a recent Cochrane rapid review that evaluated the effectiveness and safety of convalescent plasma or hyperimmune immunoglobulin transfusion in the treatment of people with COVID-19. From the eight studies it included, the review found limited and low-certainty evidence on the effectiveness and safety of convalescent plasma therapy in patients with COVID-19. The evidence was limited by the small number of participants and low-quality of included studies, as well as the inconsistency of outcome measures and reporting across studies. As African countries brace for the further spread of the virus, while exploring potential therapeutic options to mitigate its morbidity and mortality at peak, convalescent plasma transfusion may offer a therapeutic ray of hope for the continent. Considering the limited evidence of the effectiveness and safety in the treatment of COVID-19, it is imperative for this therapy to be investigated within African contexts to ascertain not only its effectiveness and safety, but also its practical implications within the capacity of national blood transfusion services and health systems in the region.

## Commentary

As the novel coronavirus continues to spread globally and across the Africa region, efforts are being accelerated to identify effective preventive and therapeutic measures to mitigate the impact of the coronavirus disease 2019 (COVID-19) [1]. While Africa is currently the least affected region globally, the COVID-19 pandemic continues to put enormous strain on the region´s frail health systems already strained by other disease burdens [2]. With no licensed vaccine or definitive treatment, management of COVID-19 has been limited to supportive care including fluid and electrolyte replacement, respiratory support and management of complications, in addition to several social distancing measures to prevent transmission [3]. Given the magnitude of the current pandemic, there have been growing interests in the investigation of the clinical benefits of convalescent plasma or hyperimmune immunoglobulin transfusion in the treatment of severely or critically ill COVID-19 patients. In this commentary, we highlight and contextualize the findings of a recently published Cochrane rapid review by Valk and colleagues, that evaluated the effectiveness and safety of convalescent plasma or hyperimmune immunoglobulin transfusion in the treatment of people with COVID-19 [4].

Convalescent plasma therapy involves the transfusion of blood plasma containing neutralising antibodies, collected from patients who recovered from an infection, to improve immune response to that infection in the recipient [3]. Hyperimmune immunoglobulin therapy is similar to convalescent plasma transfusion, but is prepared from the plasma of donors with high titres of neutralizing antibodies against a specific organism or antigen [5]. Historically, both therapies have been used in the past to treat conditions for which there were no vaccines or definitive curative treatment modalities available, with the first reports of successful treatment with passive immune therapy dating back to the early 1900s [6]. Diphtheria, pneumococcal pneumonia, hepatitis A and B, mumps, polio, measles, rabies and are conditions in which convalescent plasma has been shown to be effective [4,5]. As newer antibiotics, antivirals, and vaccines emerged, the use of convalescent serum or plasma as a frontline therapy had declined. However, with the increasing outbreak of emerging infections for which there are neither effective vaccines nor specific therapies available, modern medicine still turns to immune therapies derived from disease survivors. More recently, convalescent plasma was used during the 2009 Spanish influenza A (H1N1) and 2014-16 Ebola Virus Disease (EVD) outbreaks [6,7]. Thus, it is not surprising that convalescent plasma and hyperimmune immunoglobulin are now being considered as potential therapeutic options for the current COVID-19 outbreak [3].

In their recently published Cochrane rapid review, Valk and colleagues considered case-series, cohort, prospectively planned, and randomised controlled trials (RCTs) eligible for inclusion, if the study evaluated convalescent plasma or hyperimmune immunoglobulin for treating people with COVID-19, irrespective of disease severity, age, gender or ethnicity. Although theirs was a rapid review, the authors followed the standard Cochrane systematic review methodology and performed all steps of study screening and selection in duplicate by two independent review authors. They searched for eligible studies in multiple electronic databases up to 23 April 2020. The searched databases included the World Health Organization (WHO) COVID-19 Global Research Database, MEDLINE, Embase, Cochrane COVID-19 Study Register and the Centers for Disease Control and Prevention (CDC) COVID-19 Research Article Database. They also searched trials registries to identify on-going trials. In their search strategy, the authors combined terms for the novel coronavirus (such as SARS-CoV-2 and nCoV-2019), the disease (such as COVID-19 and COVID-19) and convalescent plasma or hyperimmune immunoglobulin (such as plasma and iimmunoglobulin) The literature search yielded a total of 1267 potentially relevant references, from which eight studies (seven case-series, one prospectively planned, single-arm intervention study) with a total of 32 participants were eventually included in the review. Of the eight included studies, seven originated from China, while one was conducted in South Korea. In addition, the authors identified 48 ongoing studies evaluating convalescent plasma (47 studies) or hyperimmune immunoglobulin (one study) in COVID-19 patients, of which 22 are RCTs. The dose, volume and timing of convalescent plasma varied widely between studies. The total volume of convalescent plasma transfused was between 200mL and 2400mL, with participants receiving between one and eight doses of plasma transfusion. Most donors were male, aged between 18 and 60 years. All donors were symptom-free and had completely recovered from COVID-19 prior to plasma donation.

The authors assessed therapeutic effectiveness by improvement of clinical symptoms (assessed by respiratory support), time to discharge from hospital, admission on the intensive care unit (ICU) and length of stay on the ICU and all-cause mortality at hospital discharge. All included studies reported some improvement in clinical symptoms in at least some participants. Time to discharge ranged from 4 to 35 days after convalescent plasma therapy. The majority of patients who were admitted in the intensive care unit (ICU) were no longer on the ICU or no longer required mechanical ventilation at the end of the reporting period. Length of stay on the ICU after receiving plasma (11 days) was reported for only on participant. In terms of safety, the review found that the majority of the included studies reported that no serious adverse events occurred following plasma transfusion; however one study reported a serious adverse event (severe anaphylactic shock). Reported non-serious adverse events included moderate fever (38.9°C). All participants were alive at the end of the reporting period, but not all participants had been discharged from hospital by the end of the study. Overall, these findings represent very limited and low-certainty evidence on the effectiveness and safety of convalescent plasma therapy for people with COVID-19, due to the low-quality of included studies; small number of participants; inconsistency of outcome measures; variation in outcome reporting across studies; and differences in the type of participants with varying severities of disease, comorbidities; and previous or concurrent treatments such as antivirals, antibiotics and corticosteroids. Moreover, the findings draw attention to the need for further research to investigate the use of convalescent plasma and hyperimmune immunoglobulin in large, randomized clinical trials or well-designed observational studies to obtain better-certainty evidence on the clinical benefits of these potential therapies for COVID-19.

While Africa currently bears the least burden of the COVID-19 pandemic, the enormous threats posed on the region´s frail health systems by the possibility of further spread of the virus are dire [2]. Therefore, African countries must brace for these threats by exploring potential therapeutic options to mitigate the morbidity and fatality of the pandemic at peak. In spite of its limited effectiveness and safety evidence in the context of COVID-19, convalescent plasma transfusion may offer a therapeutic ray of hope for the continent. Also, the previous successes in the treatment of similar respiratory diseases like the avian influenza A (H5N1), the 2009 pandemic influenza A (H1N1) and severe acute respiratory syndrome (SARS) coronavirus, convalescent plasma may offer some optimism for considering the potential benefits of the therapy against COVID-19 [6]. Like other blood components, the use of convalescent plasma or hyperimmune immunoglobulin requires functional blood transfusion resources and services. About 85% of the countries in Africa have national blood transfusion policies and services in place, albeit with varying functionality and level of development [8]. Such existing infrastructure can be leveraged for investigating and implementing plasma transfusion at population-level. There are indications that this is already beginning to happen on the continent-with the South African National Blood Service (SANBS) currently exploring ways to investigate and use the plasma for the treatment of COVID-19 patients locally [9]. However, this may not be as feasible, in other countries on the continent where national blood transfusion services are less developed with limited capacity to meet routine blood transfusion demands.

Although convalescent plasma is generally thought to be a safe and well-tolerated, adverse events can occur [10]. As with other types of blood components, the adverse events associated with plasma transfusions are well characterised. They include fever or chills, allergic reactions, and transfusion-related acute lung injury (TRALI) [4]. Critically ill patients receiving plasma transfusions are also at high risk of transfusion-associated circulatory overload (TACO), which is the leading cause of transfusion-related mortality. In addition to these, transfusion-transmitted infections, red blood cell all immunisation and haemolytic transfusion reactions have also been described following plasma transfusion [4]. As countries in the African region consider the use of convalescent plasma in the battle against this new strain of coronavirus, it is prudent to ensure that there are system-wide resources and infrastructure to foster the efficient and safe transfusion of plasma. During the 2014 Ebola outbreak, attempts to use convalescent plasma brought revealed the challenges faced by African countries in ensuring safe blood collection and transfusion [8]. Therefore, building local capacity of African blood transfusion sub-systems in donor recruitment, blood collection, adequate blood screening and intra-transfusion safety monitoring will be crucial for the safe and rational use of convalescent plasma in the region [8]. It is also important for further research to investigate the clinical benefits and practicality of convalescent plasma or hyperimmune immunoglobulin in African contexts. These can be assessed through trials or well-designed observational studies as countries implement plasma therapy in their COVID-19 treatment settings. Such research efforts are needed to establish better-certainty evidence on the clinical benefits of these potential therapies for informing COVID-19 treatment approaches in the region.

## Conclusion

From the highlighted Cochrane review, there is limited and low-certainty evidence on the effectiveness and safety of convalescent plasma or hyperimmune immunoglobulin therapy for people with COVID-19, due to small sample sizes and study design limitations of the included studies. African countries may consider further investigation to ascertain not only the effectiveness and safety but the practicality of these therapeutic options within the capacity of their national blood transfusion services.

## Competing interests

The authors declare no competing interests.
